# Supplementary material for: Clinicopathological Profiles Associated with Discordant RAS Mutational Status between Liquid and Tissue Biopsies in a Real-World Cohort of Metastatic Colorectal Cancer
Source: Cancers (Basel). 2023 Jul 12;15(14):3578. doi: 10.3390/cancers15143578 (PMC10377339; doi:10.3390/cancers15143578)
Supplement: Supplementary file 1 [file cancers-15-03578-s001.zip › cancers-2464769-supplementary.pdf]

Supplementary Materials

Table S1. Concordance of *RAS* mutational status in patients with synchronous mCRC treated with tumour resection before the liquid biopsy extraction.

	Nº	TP	TN	FP	FN	Concordance[1] 95% CI	Sensitivity [2] 95% CI	Specificity [3] 95% CI	K [4] 95% CI	OR 95% CI	*p*-value
Not tumour resection											
	122	60	50	6	6	90.2<br>83.6-94.3	90.9<br>80.6-96.3	89.3<br>77.4-95.6	0.80<br>0.70-0.91		
Primary tumour resection											
	77	39	28	2	8	87.0<br>77.7-92.8	83.0<br>68.7-91.9	93.3<br>76.5-98.8	0.74<br>0.58-0.89	1.4<br>0.7 - 2.7	0.33
Primary tumour and metastases resection											
	17	8	6	0	3	77.7<br>54.8-91.0	72.7<br>39.3-92.7	100.0<br>51.7-100.0	0.65<br>0.30-1.0	2.1<br>0.5 – 8.2	0.49

Table S1 includes information from 216 patients with synchronous metastases. Resection of Metastatic tumour tissue was performed in 8.33% of patients.True positive TP: Tissue *RAS* mutated/ctDNA *RAS* mutated; True negative TN: Tissue *RAS* wt/ctDNA *RAS* non-mutated; False positive FP: Tissue *RAS* wt/ctDNA *RAS* mutated; False negative FN: Tissue *RAS* mutated/ctDNA *RAS* non-mutated[1] Overall percentage agreement (95% Confidence Intervale); [2]Positive percentage agreement (95% Confidence Intervale); [3]Negative percentage agreement (95% Confidence Intervale); [4] Cohen's kappa coefficient (95% Confidence Intervale); [5] odds ratio (95% confidence interval)

Table S2. Effect of the resection of the primary tumour in the concordance of *RAS* mutational status of patients with synchronous metastases.

	Nº	TP	TN	FP	FN	Concordance[1] 95% CI	Sensitivity[2] 95% CI	Specificity [3] 95% CI	K [4] 95% CI
Not tumour resection									
Left	40	18	19	1	2	92.5 80.1-97.4	90.0 66.9-98.2	95.0 73.1-99.7	0.85 0.69-1.0
Rectum	57	29	24	3	1	93.0 83.3-97.2	96.7 80.9-99.8	88.9 69.7-97.1	0.86 0.73-0.99
Right	19	10	6	2	1	84.2 62.4-94.5	90.9 57.1-99.5	75 35.6-95.5	0.67 0.33-1.0
Tumour resection									
Left	47	19	23	2	3	89.4 77.4-95.4	86.4 64.0-96.4	92.0 72.5-98.6	0.79 0.61-0.96
Rectum	15	9	4	0	2	86.7 62.1-96.3	81.8 47.8-96.8	100 39.6-100.0	0.71 0.33-1.0
Right	29	17	6	0	6	79.3 61.6-90.2	73.9 51.3-88.9	100 51.7-100.0	0.54 0.21-0.87

Table S2 includes information from 216 patients with synchronous metastases. Rsection of the Primary tumour was performed in 43.5% of patients. True positive TP: Tissue *RAS* mutated/ctDNA *RAS* mutated; True negative TN: Tissue *RAS* wt/ctDNA *RAS* non-mutated; False positive FP: Tissue *RAS* wt/ctDNA *RAS* mutated; False negative FN: Tissue *RAS* mutated/ctDNA *RAS* non-mutated.[1] Overall percentage agreement (95% Confidence Intervale); [2]Positive percentage agreement (95% Confidence Intervale); [3]Negative percentage agreement (95% Confidence Intervale); [4] Cohen's kappa coefficient (95% Confidence Intervale)

.

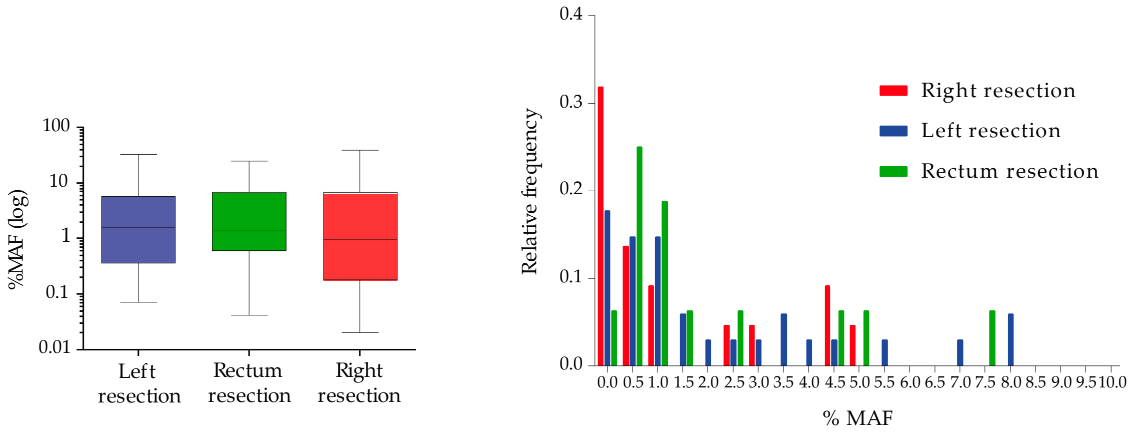

(a) **(b)**

Figure S1. Impact of the resection of the primary tumour on the detection of plasmatic *RAS* mutations. (a) Plasmatic *RAS* MAF percentage in patients treated with tumour resection. Data included patients with synchronous and metachronous metastases. Blox-Plots represent the 75th and 25th interquartile range. Y axe is log scale. X axe represents: Blue: ctDNA *RAS* MAFs from patients with mCRC originating in the left colon. Green: ctDNA *RAS* MAFs from patients with primary tumour located in the rectum. Red: ctDNA *RAS* MAFs from patients with right-sided mCRC. (b) Relative frequency distribution of the % MAFs in patients with mCRC originating in different sites of the colon and treated with tumour resection. Y axe represents the relative frequency of the %MAF in each mCRC population. X axe represents Bin centre. Bin width is of 0.05. Bin 1 includes all % MAFs below 0.25%. Only MAFs below 10% are plotted in the graph
